# Massage and the Original Swedish Movements

**Published:** 1899-11

**Authors:** 


MASSAGE AND THE ORIGINAL SWEDISH MOVEMENTS. Their application to various diseases of the body. Lectures before the training schools for nurses connected with the Hospital of the University of Pennsylvania, German Hospital, Woman's Hospital, etc. By KURRE W. OSTROM, from the Royal University of Upsala, Sweden. Fourth edition, revised and enlarged, with 105 illustrations. Philadelphia: P. Blakiston's Son & Co. 1899.

Ostrom is one of the authorities on massage and the Swedish movements. His book has passed through four editions in less than ten years. The work is practical in character, and will be of value to the physician who desires some knowledge of mechano-therapy, and of especial interest to the nurse and professional masseur.

